# Audit of screen failure in 15 randomised studies from a low and middle-income country

**DOI:** 10.3332/ecancer.2022.1476

**Published:** 2022-11-23

**Authors:** Deevyashali Parekh, Vijay M Patil, Kavita Nawale, Vanita Noronha, Nandini Menon, Sucheta More, Supriya Goud, Srushti Jain, Vijayalakshmi Mathrudev, Zoya Peelay, Sachin Dhumal, Shweta Jogdhankar, Kumar Prabhash

**Affiliations:** Department of Medical Oncology, Tata Memorial Centre and HBNI, Parel, Mumbai 400012, India; † These authors contributed equally

**Keywords:** clinical trials, statistics, head and neck cancer, oncology, epidemiology, solid tumour

## Abstract

**Background:**

Growth and development in patient management occurs via randomised studies. Screen failure is a significant hurdle while conducting randomised studies. There is limited data available from low and middle-income countries about factors resulting in screen failure. Hence, this audit was performed to identify the proportion of patients who screen failed and to elucidate reasons for the same.

**Methods:**

This was an audit of 15 randomised studies performed by medical oncology solid tumour unit II of Tata Memorial Centre. The screening logs of these studies were acquired. From the screening logs, data regarding the number of patients who had screen failed & reason for the same were obtained. Descriptive statistics were performed.

**Results:**

A total of 7,481 patients were screened for 15 randomised clinical studies. Out of these, 3,666 (49.0%) patients were enrolled into trials and 3,815 (51.0%) screen failed. The most common reason for screen failure was ‘not meeting inclusion criteria’ (54.9%) followed by declining to take treatment (22.2%). Other factors that affect enrolment were ‘not willing to stay in the locality of the trial site’ (6.2%), being recruited in other studies (3.7%), poor performance status (PS) (3.4%), non-compliance (2.2%), meeting exclusion criteria (0.9%) and ‘other’ (6.5%).

**Conclusion:**

The commonest causes of screen failure in lower and middle-income countries are non-meeting of inclusion criteria followed by declining to take treatment, not willing to stay in locality of trial site, recruited into other studies, poor PS, non-compliance, meeting exclusion criteria & ‘other’. This information would help analysing and hence planning of newer strategies to decrease the rate of screen failure.

## Introduction

Randomised clinical studies are fundamental to improvement and development in patient management. They help evaluate the efficacy of an existing or new intervention against standard of care or placebo hence offering valuable information on the relative benefit of the intervention. While a single study cannot provide conclusive evidence standalone, each such study helps lend credence to a hypothesis regarding the efficacy of an intervention, reduces bias and might provide additional data of idiosyncrasies in efficacy by disease type, severity, ethnicity, age, gender, etc. Thus, randomised clinical trials (RCTs) are often considered a robust way to determine the cause–effect relationship between an intervention and a given outcome [1].

Screen failure occurs when a patient is screened to be enrolled into a given study or trial but is unable to be enrolled. Screen failure is a significant hurdle while conducting randomised studies. Patients that are screened are already from a narrow set of patients deemed as potentially suitable for that trial and thus screen failure leads to a loss of valuable data in that trial. The power of a trial correlates inversely with the sample size and hence screen failure negatively impacts this. Screen failure also leads to a loss of valuable time and resources in the screening process which ultimately do not contribute towards the outcome result and often also in continued trial recruitment.

There is limited data available from low and middle income country (LMIC) about factors resulting in screen failure. Thus, we decided to perform this audit to try to identify the number of patients who underwent screen failure and attempt to elucidate reasons for the same.

## Method

### Study conduct

This single-centre retrospective analysis was done at our institution in India. Fifteen RCTs that were performed at our institution were audited. The study was conducted in accordance with the standards laid down by the declaration of Helsinki, International Conference on Harmonisation Good Clinical Practice (ICH-GCP) and Indian Council of Medical Research (ICMR). Data collection for the study was done in 2022.

### Patient selection

**Figure 1.**
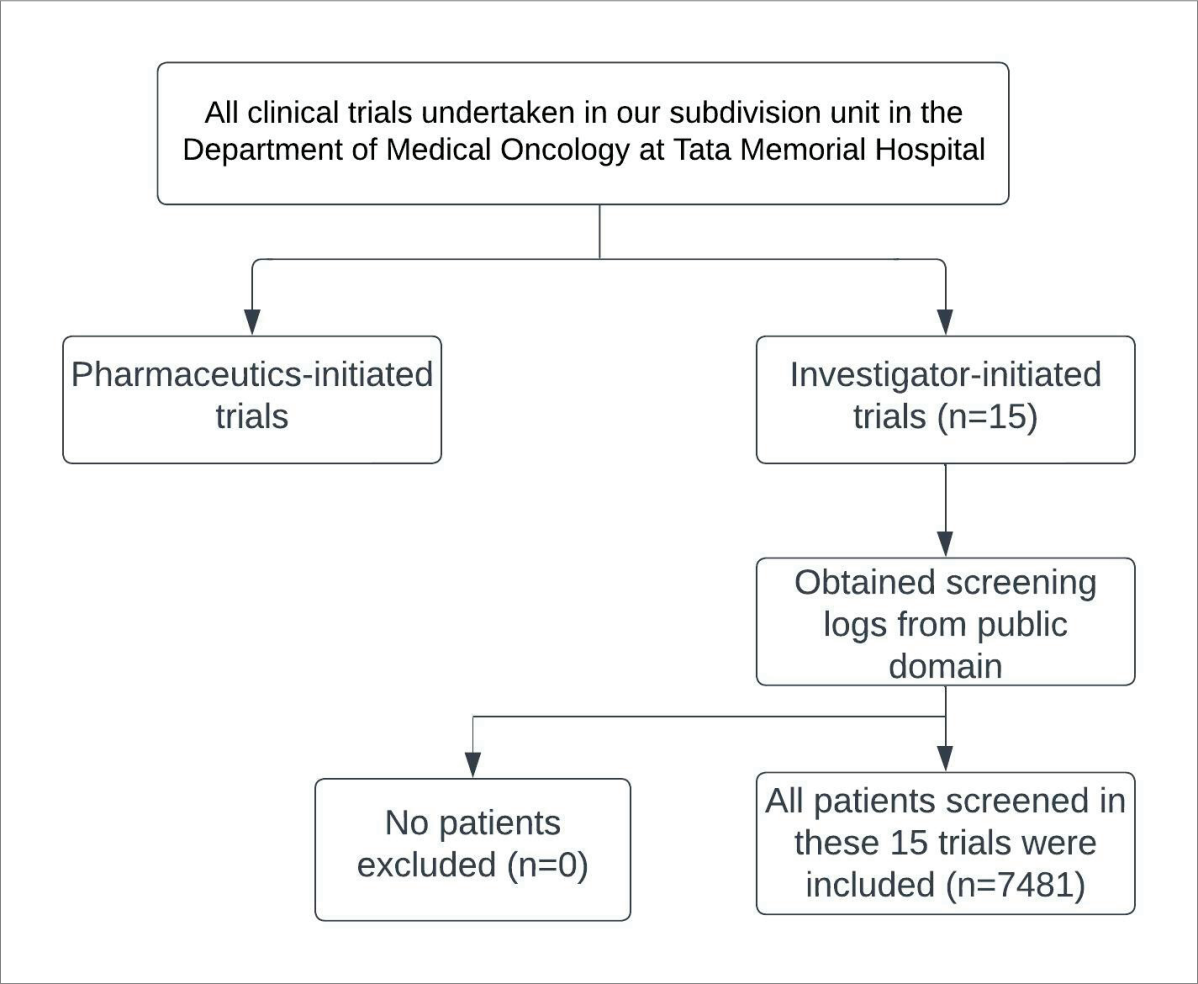
Flowchart of inclusion and exclusion criteria for patients in this study.

Patients from 15 randomised studies done at our institution that underwent screen failure were selected for audit in this study. Screening was done by investigators in each study. Patients who were deemed as potential fits for each respective study underwent a screening procedure to determine enrolment into that study. If a patient was unable to be enrolled into the study for any given reason after being screened, they were classified as ‘screen failure’. A flowchart of inclusion and exclusion criteria for patients in this study is depicted in Figure 1.

### Data collection

The data was collected on a predefined data collection sheet which was shared with all investigators. The data collected was of the number of patients screened for each of the 15 trials, the number of patients who screen failed for each trial and the reason for their screen failure.

## Results

**Figure 2.**
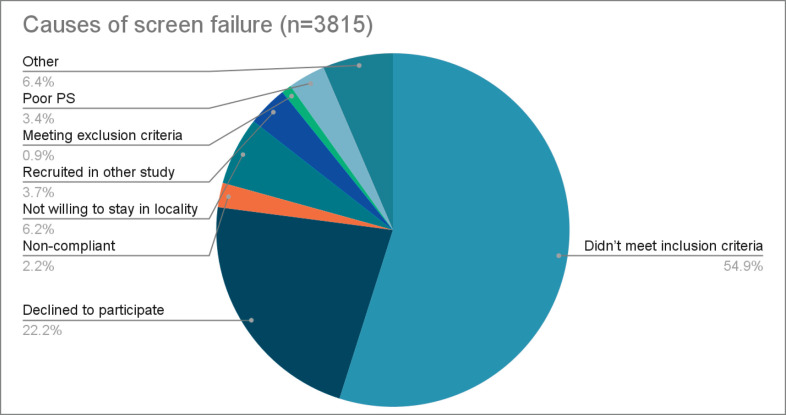
Pie-chart showing the distribution of causes of screen failure (*n* = 3,815).

A total 7,481 patients were screened for 15 randomised clinical studies. Out of these, 3,666 (49.0%) patients were enrolled into trial and 3,815 (51.0%) screen failed (Table 1). Details of recruitment, intent and targeted site for each of the 15 studies are given in Table 2. Further details including the title as well as a ‘key points’ summary of each of these trials is included in Table S1 in the Supplementary Appendix. Total, enrolled and screenfailed patients by therapeutic site of trial and by intent of trial are depicted in Table 3 and 4 respectively. The most common reason for screen failure was non-meeting inclusion criteria (54.9%) followed by declining to take treatment (22.2%). These were followed by ‘Other’ (6.4%) which included reasons like taking a certain treatment at a different hospital or death before being able to complete screening. Not willing to stay at locality of trial site (6.2%), recruited in other study (3.7%) and poor performance status (PS) were the other significant causes for screen failure listed in order of magnitude. The full list of causes for screen failure is listed in Table 5 and in Figure 2.

## Discussion

Randomised clinical studies are the cornerstone of advancement and development in patient management. They help evaluate the efficacy of an existing or new intervention against standard of care or placebo hence offering valuable information on the relative benefit of the intervention. RCTs are often considered a robust way to determine a cause–effect relationship between an intervention and a given outcome [1].

Screen failure occurs when a patient is screened to be enrolled into a given study or trial but is unable to be enrolled. Screen failure is a significant hurdle while conducting randomised studies [2].

Our data shows that the largest determinant of screen failure for patients is ‘not meeting the inclusion criteria’ (54.9%). While well-framed, objective criteria maintain the integrity of a study, this finding suggests that increasingly strict or rigid criteria could be contributing to a large number of screen failures [3, 4]. Due to the burden of a large number of screen failures, it may be worth looking into and analysing inclusion criteria to ensure a balance between maintaining integrity of data and not being overly rigid [5–7].

Declining to participate in the trial (22.2%) and non-compliance (2.2%) made up a big part of screen failure. These are especially important determinants as they seem most amenable to correction [8, 9]. To an extent, non-compliance of patients and also their decision against participating in the trial can benefit from better counselling and teaching methods by investigators involved in recruitment. Care should be taken to explain treatment options and regimens in a language that the patient understands and is comfortable with. The teach-back method can be used to ensure patient understanding. Involved personnel can undergo some training in counselling practices with the hope that it may lead to greater patient participation in trials.

The other criteria making a significant contribution that has room to be benefitted is ‘unwillingness to stay in the locality of trial site’ (6.2%). Treatment and trial participation often require regular treatment at short intervals and follow-up investigations at short intervals. Trials of this nature would require that the patient have ease of geographic access to the site of the trial on a daily or weekly basis. For some patients, the financial, logistical and social challenges of relocating to the trial site are a significant hurdle. Trials of relatively uncommon conditions designed with such accessibility requirements could have funding and provision allowing the relocation of a patient and their support to the site of the study temporarily. It could also provide basic necessities and/or a stipend, travel arrangement, concession, etc.

Screen failure has a detrimental effect on trial power because sample size and power are inversely correlated. Failure to pass a screening also wastes time and money that could have gone toward improving the outcome, and it frequently results in trial recruitment continuing [10, 11].

There is limited data available from LMICs about factors resulting in screen failure. A similar audit coming out of a LMIC showed the screen failure rate at an impressive 5% [12, 13].

Thus, we decided to perform this audit to try to identify the number of patients who underwent screen failure and attempt to elucidate reasons for the same.

## Conclusion

This audit was performed as limited data exists on factors impacting screen failure in trials in LMICs. A total 7,481 patients were screened for 15 randomised clinical studies. Out of these, 3,666 (49.0%) patients were enrolled into trial and 3,815 (51.0%) screen failed. The most common reason of screen failure was non-meeting inclusion criteria (54.9%) followed by declining to take treatment (22.2%). This information would help analyse and hence planning of newer strategies to decrease the rate of screen failure.

## Key findings

This audit was performed as limited data exists on factors impacting screen failure in trials in LMICs.

A total of 7,481 patients were screened for 15 randomised clinical studies. Out of these, 3,666 (49.0%) patients were enrolled into trial and 3,815 (51.0%) screen failed.

The most common reason of screen failure was non-meeting inclusion criteria (54.9%) followed by declining to take treatment (22.2%).

This information would help analyse and hence planning of newer strategies to decrease the rate of screen failure.

## Conflicts of interest

None.

## Funding statement

No funding was obtained to perform this study.

## Figures and Tables

**Table S1.** Details of each study included in this audit.

Sr no	Name	Recruiting From	Recruiting till	Site	Intent	About the paper
1	Phase 2 going to phase 3 randomized study for evaluation of nivolumab and metronomic as palliative therapy in head and neck cancer.	Jan-21	Sep-21	Head and Neck	Palliative	In our previous study, metronomic chemotherapy (MC) improved survival in this setting. Retrospective data suggest that a low dose of nivolumab may be efficacious. Hence, we performed this study to assess whether the addition of low dose nivolumab to MC improved the overall survival. This was a randomised phase 3 superiority open-label study. 208 patients were screened, 57 patients screen failed and 151 patients were enrolled.
2	MACE-CTRT : Metronomic Adjuvant Chemotherapy Evaluation in locally advanced head and neck cancers post radical chemoradiation	Nov-16	Aug-19	oropharynx ,larynx, hypopharynx	Curative	The current options produce unsatisfactory outcomes in locally advanced head and neck cancer. Addition OMCT seems to improve survival when administered as adjuvant treatment in locally advanced head and neck cancers in multiple retrospective studies. This therapy is associated with no major grade 2-4 toxicity. Hence we have planned this study to assess the efficacy of adjuvant OMCT (consisting of weekly methotrexate and celecoxib) in locally advanced head and neck cancers post radical chemoradiation.This is Phase3 randomized controlled trial performed at Tata Memorial centre. We have enrolled 137 patients in this study form 2016 to 2019.
3	DHANUSH : Docetaxel as radiosensitizer in Head And Neck cancer patients,Unsuitable for cisplatin based chemoradiation	Jul-17	May-21	Head and Neck	Palliative	There is a lack of published literature on systemics therapeutic options in cisplatin-ineligible locally advanced head and neck squamous cell carcinoma (LAHNSCC) patients undergoing chemoradiation. In this study, Docetaxel was assessed as a radiosensitizer in this situation. This was a phase 2 going to phase 3 randomized, parallel, open-label, single center and superiority study. 777 patients were screened, 421 patients screen failed while 356 patients were enrolled.
4	Once-a-Week Versus Once-Every-3-Weeks Cisplatin Chemoradiation for Locally Advanced Head and Neck Cancer: A Phase III Randomized Noninferiority Trial	2013	2017	Head and Neck	Curative	In this phase III randomized trial, we assessed the noninferiority of cisplatin 30 mg/m2 given once a week compared with cisplatin 100 mg/m2 given once every 3 weeks, both administered con-currently with curative intent radiotherapy in patients with LAHNSCC. Between 2013 and 2017, we screened 892 patients, 592 screen failed and 300 patients were enrolled, 150 to each arm.
5	Diclofenac versus tramadol for mucositis related pain in head and neck cancer patients undergoing concurrent chemoradiation—a phase 3 study	Apr-17	May-19	Oral Oropharynx Larynx Hypopharynx Others	Palliative	This study was designed to compare the analgesic effect of a non-steroidal anti-inflammatory drug (diclofenac) versus a weak opioid (tramadol) on oral mucositis related pain during CTRT in head and neck cancers. It was an open-label, parallel design, superiority randomised controlled study. In this study, head and neck cancer patients undergoing radical or adjuvant chemoradiation were randomly assigned to either diclofenac or tramadol for mucositis related pain control. The primary endpoint was analgesia after the first dose. The secondary endpoints were the rate of change in analgesic within 1 week, adverse events and quality of life. 308 patients were screened, 180 screen failed while 128 were enrolled.
6	A Randomized Phase 3 Trial Comparing Nimotuzumab Plus Cisplatin Chemoradiotherapy Versus Cisplatin Chemoradiotherapy Alone in Locally Advanced Head and Neck Cancer	2012	2018	oropharynx, larynx, hypopharynx, or oral cavity	Curative	Because the addition of nimotuzumab to chemoradiation in patients with locally advanced head and neck cancer improved outcomes in a phase 2 study, the authors conducted a phase 3 study to confirm these findings. It was an open-label, investigator-initiated, phase 3, randomized trial conducted from 2012 to 2018. 754 patients were screened, 536 patients enrolled and 218 scree-failed. Adult patients with locally advanced head and neck cancer who were fit for radical chemoradiation were randomized 1:1 to receive either radical radiotherapy (66-70 grays) with concurrent weekly cisplatin (30 mg/m^2^) (CRT) or the same schedule of CRT with weekly nimotuzumab (200 mg) (NCRT).
7	Low-cost oral metronomic chemotherapy versus intravenous cisplatin in patients with recurrent, metastatic, inoperable head and neck carcinoma: an open-label, parallel-group, non-inferiority, randomised, phase 3 trial	May-16	Jan-20	Head and Neck	Palliative	In a previous phase 2 study, patients with head and neck cancer who received metronomic chemotherapy had better outcomes when compared with those who received intravenous cisplatin, which is commonly used as the standard of care in LMICs. We aimed to do a phase 3 study to substantiate these findings. It was an open-label, parallel-group, non-inferiority, randomised, phase 3 trial at the Department of Medical Oncology, Tata Memorial Center, Homi Bhabha National Institute, Mumbai, India. We randomly assigned (1:1) participants to receive either oral metronomic chemotherapy or intravenous cisplatin once every 3 weeks for six cycles Between May 16, 2016, and Jan 17, 2020, 594 patients were screened, 172 screen-failed and 422 patients were enrolled.
8	Aprepitant for Cough Suppression in advanced lung cancer	2017	2018	Lung	Palliative	Patients with advanced lung cancer and cough lasting over 2 weeks despite a cough suppressant were randomized 1:1 to aprepitant 125 mg orally on day 1 and then 80 mg orally on days 2 to 7 with physician’s choice of antitussive; or to physician’s choice of antitussive alone. Between 2017 and 2018, 237 patients were screened, 109 screen failed and 128 patients enrolled.
9	Randomized phase 3 open label study of quality of life of patients on Pemetrexed versus Erlotinib as maintenance therapy for advanced non squamous non EGFR mutated non small cell lung cancer	Nov-14	Mar-17	Lung	Palliative	The study compared pemetrexed maintenance with erlotinib maintenance in non-squamous non Epidermal Growth Factor Receptor (EGFR) mutated non-small cell lung cancer (NSCLC). The study was an open label, single centre, parallel, phase 3 randomized study with 1:1 randomization between maintenance pemetrexed arm and erlotinib arm. 351 patients were screened, 151 patients screen failed and 200 patients were enrolled.
10	Phase III Non-inferiority Study Evaluating Efficacy and Safety of Low Dose Gemcitabine Compared to Standard Dose Gemcitabine With Platinum in Advanced Squamous Lung Cancer	May-13	Mar-18	Lung	Palliative	Prolonged infusion of low dose gemcitabine (PLDG) in combination with platinum has shown promising activity in terms of improved response rate and progression free survival (PFS); especially in squamous non-small cell lung cancer (NSCLC). Hence, we conducted a phase 3 randomized non-inferiority study with the primary objective of comparing the overall survival (OS) between PLDG and standard dose of gemcitabine with platinum. 521 patients were screened, 213 patients screen failed while 308 patients were enrolled.
11	Gefitinib Versus Gefitinib Plus Pemetrexed and Carboplatin Chemotherapy in *EGFR*-Mutated Lung Cancer	2016	2018	Lung	Palliative	It was theorised that adding pemetrexed and carboplatin chemotherapy to an oral tyrosine kinase inhibitor may improve outcomes in EGFR-mutant advanced non-small-cell lung cancer (NSCLC). This was a phase III randomized trial in patients with advanced NSCLC harboring an EGFR-sensitizing mutation and a performance status of 0 to 2 who were planned to receive first-line palliative therapy. Between 2016 and 2018, 712 patients were screened, 362 patients screen failed and 350 patients were randomly assigned to Gef (n = 176) and Gef+C (n = 174).
12	Phase III study of gefitinib or pemetrexed with carboplatin in EGFRmutated advanced lung adenocarcinoma	Feb-12	Apr-16	Lung	Palliative	Oral tyrosine kinase inhibitor has been shown to prolong progression-free survival (PFS) in epidermal growthfactor receptor (EGFR) mutation positive adenocarcinoma; however, the comparator arm has not included the current standard adenocarcinoma and patients from Indian subcontinent. This was an open-labelled, randomised, parallel group study comparing gefitinib (250 mg orally daily) with pemetrexed (500 mg/m2) and carboplatin (area under the curve 5) doublet intravenous induction chemotherapy regimen followed by maintenance pemetrexed (500 mg/m2) in patients with EGFR-activating mutation-positive stage IIIB or stage IV adenocarcinoma lung in the first-line setting in Indian population.
	Phase III randomized trial comparing intravenous to oral iron in patients with cancer-related iron deficiency anemia not on erythropoiesis stimulating agents	Mar-10	Mar-15	Any	Palliative	We aimed to find the optimal route of iron supplementation in patients with malignancy and iron deficiency (true or functional) anemia not receiving erythropoiesis stimulating agents (ESA). Adult patients with malignancy requiring chemotherapy, hemoglobin (Hb) <12 g/dL and serum ferritin <100 mcg/mL, transferrin saturation <20% or hypochromic red blood cells >10% were randomized to intravenous (IV) iron sucrose or oral ferrous sulfate. 749 patients were screened, 557 patients screen failed and 192 patients were enrolled.

**Table 1.** Number of patients for each screening outcome of the total screened patients from 15 RCTs. RCT, Randomised controlled trial.

Total number of patients screened in 15 RCTs, *N* = 7,481		
Screening outcome	Number of patients (No.)	Percentage of total patients (%)
Enrolled	3,666	49.0
Screen failed	3,815	51.0

**Table 2.** Baseline characteristics and recruitment overview of all 15 RCTs included in this study.

Sr. no.	Recruitment		Intent	Site	Screened	Enrolled	Screen failed
	From	Till					
1	01/2021	09/2021	Palliative	Head and neck	208	151	57
2	11/2016	08/2019	Curative	Head and neck	637	137	500
3	07/2017	05/2021	Curative	Head and neck	777	356	421
4	2013	2017	Curative	Head and neck	892	300	592
5	04/2017	05/2019	Curative	Head and neck	308	128	180
6	2012	2018	Curative	Head and neck	754	536	218
7	05/2016	01/2020	Palliative	Head and neck	594	422	172
8	2017	2018	Palliative	Lung	237	128	109
9	11/2014	03/2017	Palliative	Lung	351	200	151
10	05/2013	03/2018	Palliative	Lung	521	308	213
11	2016	2018	Palliative	Lung	712	350	362
12	02/2012	04/2016	Palliative	Lung	497	290	207
13	03/2010	03/2015	Curative	Any	749	192	557
14	01/2017	03/2017	Curative	Brain	112	65	47
15	03/2018	01/2019	Palliative	Brain	132	103	29

**Table 3.** Total, enrolled and screen-failed patients by major therapeutic area of trial.

Major therapeutic area	Intent	Total patients screened (*n*)	Number of patients who were enrolled (%)	Number of patients who screen-failed (%)
Head and neck	Palliative	802	573 (71.4)	229 (28.6)
	Curative	3,368	1,457 (43.3)	1,911 (56.7)
	Total	4,170	2,030 (48.7)	2,140 (51.3)
Lung	Palliative	2318	1,276 (55.0)	1,042 (45.0)
	Curative	0	0 (0)	0 (0)
	Total	2,318	1,276 (55.0)	1,042 (45.0)
Brain	Palliative	132	103 (78.0)	29 (22.0)
	Curative	112	65 (58.0)	47 (42.0)
	Total	244	168 (68.9)	76 (31.1)
Any	Palliative	0	0 (0)	0 (0)
	Curative	749	192 (25.6)	557 (74.4)
	Total	749	192 (25.6)	557 (74.4)

**Table 4.** Total, enrolled and screen-failed patients by intent of trial.

Intent	Total patients screened (*n*)	Number of patients who were enrolled (%)	Number of patients who screen-failed (%)
Palliative	3,252	1,952 (60.0)	1,300 (40.0)
Curative	4,229	1,714 (40.5)	2,515 (59.5)

**Table 5.** Reason for screen failure. PS, Performance status.

Total number of patients who underwent screen failure, *N* = 3,815			
Cause for screen failure	Number of patients (No.)	Percentage of patients who screen failed (%)	Percentage of all screened patient (%)
Didn’t meet inclusion criteria	2,094	54.9	28.0
Declined to participate	846	22.2	11.3
Non-compliant	85	2.2	1.1
Not willing to stay in locality of trial site	238	6.2	3.2
Recruited in other study	141	3.7	1.9
Meeting exclusion criteria	35	0.9	0.5
Poor PS	130	3.4	1.7
Other	246	6.5	3.3
